# ERP Study of Mine Management System Warning Interface under Fatigue

**DOI:** 10.3390/ijerph191912616

**Published:** 2022-10-02

**Authors:** Yuxin Bai, Jiang Shao, Ying Zhang, Lulu Chen, Xijie Zhao, Fangyuan Tian, Chengqi Xue

**Affiliations:** 1 School of Architecture & Design, China University of Mining and Technology, Xuzhou 221116, China; 2 School of Safety Engineering, China University of Mining and Technology, Xuzhou 221116, China; 3 School of Management, Xi’an University of Science and Technology, Xi’an 710054, China; 4 School of Mechanical Engineering, Southeast University, Nanjing 211189, China

**Keywords:** fatigue, mine management system, early-warning interface, event-related potential

## Abstract

Due to the large volume of monitoring data in mines, concentrating on and reviewing the data for a long period of time will easily cause fatigue. To study the influence of different visual codes of early-warning interfaces on the response of individuals who are fatigued, the changes in the subjective fatigue and corresponding frequency waves are compared before and after a fatigue-inducing task, as well as using event-related potential to study the behavioral data and EEG signals of subjects who participated in an oddball task on an early-warning interface. The results showed that all 14 subjects became fatigued after the fatigue-inducing task, and the amplitude of P200 when text is used in a fatigued state was the largest, with the longest latency. The subjects showed a slower reaction time and a reduced accuracy rate, thus indicating that in designing a warning interface, when text rather than color is used as a visual code, the operating load will be larger, mental load is increased, and attention resources are consumed. The experimental results provide the basis for the design and evaluation of early-warning interfaces of mine management systems.

## 1. Introduction

Mining accidents are considered a national public emergency in China [1], so the monitoring of safety conditions in mines is an important part of managing accident and disaster emergencies. China has enjoyed relative success in managing mining safety conditions. In 2021, there were 356 mine-related accidents and 503 mine-related deaths, which is a decrease of 68 and 73, or 16% and 12.7% from the previous year, respectively. The decrease is informed by experience, which shows that standardizing the safety management systems of mines and preventing sudden mining accidents and addressing these accidents in a timely manner can minimize casualties and property losses, ensure the wellbeing of residents and properties, and maintain social stability [2]. The 14th Five-Year Plan (2021–2025) for the National Economic and Social Development and Vision 2035 of the People’s Republic of China proposes that improving national emergency management systems is an important component of considering both development and security and building a safer China with an enhanced capacity to prevent and control disasters. The monitoring by mining management systems can provide insights into a more accurate future vision in a multitude of directions [3], facilitate intelligent scheduling, control risks, predict hazards, and provide advanced warning of issues. However, there are a large number of mining areas in China. They form a pattern that is widely distributed and tightly clustered. Additionally, mine production can be a complex process [4]. To better monitor mines for safety purposes, the monitored areas are generally equipped with a large number of monitoring devices. They gather a large volume of vague information, which means that managers who try to stay focused to analyze the obtained data for a long period of time will be mentally exhausted with reduced work efficiency.

In general, the early-warning mechanism of mine management systems monitors any risky behaviors, hazards, and dangerous environmental factors [5]. Identifying safety violations in a timely manner is important. There must be early warning of risks and elimination of the potential areas for mishaps to effectively ensure the safety of a mine. Existing studies have shown that an improperly designed early-warning interface is detrimental and leads to the escalation of many accidents [6]. Only by ensuring an optimal early-warning interface design will managers be able to quickly and accurately respond to the problem, even in a fatigued state. In comparison to the coding of visual information of other digital interfaces, there are few studies that focus on the early-warning interface design of mine management systems. In interactive interfaces, the most commonly coded visual information includes color [7], size [8], and shape [9], among which the interface information display methods are mainly divided into those that use text and color as the visual codes. For instance, Rolke et al. used event-related potential (ERP) to study the mechanism that influences the different colors and shapes as visual codes in visual information processing [10]. Yeh et al. found that stimuli with large color differences had shorter P100 latencies and larger P300 amplitudes [11]. Bayliss found that P300 was induced by red light, but not by yellow light [12]. There are relatively few studies on the P200 compared to the P300. Chen et al. found that the greater the correlation between product color and image, the more significant the P200 component [13]. Xue et al. found that N100 and P200 have more significant changes in color matching through ERP research on interface color matching [14]. Comparing the different amplitudes of the P200, Liu et al. found that color features required more attention resources than shape features [15]. In the ERP study, it is found that high-level cognitive activities may induce more P200, N400, and LPC components, of which the P200 component may be more related to the text [16]. Perne et al. found that compared to unknown categories (e.g., Asiatic characters), over-learned stimuli (e.g., letters) caused a shorter P2 latency [17]. Barber et al. found that high-density words trigger a smaller P200 than low-density words [18].

The P200 is a component of ERP which is evoked at the early stages. P200 appears at around 100–200 ms after the stimulus is presented, and the main evoked potential is located in the frontal lobe [19]. P200 is also associated with attention to stimuli in the early stages, thus reflecting the processing activity of stimuli [20,21]. The latency and amplitude of P200 may correlate with aspects such as selective attention or stimulus-encoding processes [22]. The P200 amplitude embodies an instinctive response of the brain to a stimulus and reflects early rapid automatic processing [23], with a higher wave amplitude representing that stimulus would consume more attentional resources [24]. Jin et al. found that a higher P200 amplitude shows that the subject is paying more attention to the stimuli, and more attention resources are mobilized [25]. Pernet et al. found that P2 and N2 were affected by visual stimulus categories [17]. Lee et al. proposed that P200 may be related to anxiety, negative emotions, and fatigue [26]. Mun et al. found that mental fatigue caused by mobile three-dimensional (3D) viewing causes a decrease in the amplitude of the P200 and LPP [27]. Sun et al. found significant differences in the mean amplitude of P200 before and after brain fatigue [28]. The more familiar the subject is with the stimulus, the less cognitive resources are consumed, and the longer P200 latency to unfamiliar stimuli. In addition to this, P200 is also associated with task difficulty, and Miller et al. found that P2 amplitude is inversely related to task difficulty and decreases as task difficulty increases [29,30].

At present, the ERP technique has become an intuitive and effective means in psychology, cognitive neuroscience, and ergonomics to reflect the cognitive processing of information, such as decision-making that involves risk. Moore et al. used ERP in an experiment with 14 subjects and found that mental fatigue can inhibit cognitive activity based on a listening task [31]. The fatigue increases over time, which results in a decline in attention paid to the task. Dimitrakopoulos et al. also experimentally confirmed that mental fatigue was found in their study with 40 subjects who performed 2 driving tasks [32]: one that was low intensity with a longer duration, and one that was higher in intensity but shorter in duration. The subjects showed a decline in cognitive tasks before and after conducting the tasks, as well as disruptions in processing information due to fatigue. Although previous studies such as those by Zhang et al. and Huang et al. also used ERP for safety analysis to determine how risk is perceived and decisions are made while fatigued in the coal mining context [33,34], there are still very few studies on the risk perception and decision-making of managers as the focus. Moreover, at present, research work on emergency responses in mines in China mostly focuses on the strategic construction of a macro-management system, and few in the literature have evaluated and optimized an early-warning interface design in managing the operations of a mine from the perspective of cognitive science and neuroscience. Therefore, this paper uses the electroencephalography (EEG) data of subjects before and after a fatigue-inducing task to conduct a comparative study. Subjective evaluation, user behavior performance, and ERP are used to analyze the characteristics of EEG that indicate that the subjects are in a fatigued state. The early-warning interface design provides the basis to improve the work performance.

## 2. Materials and Methods

### 2.1. Subject Recruitment 

A total of 14 undergraduate and postgraduate students (7 males and 7 females, mean age = 21.857, SD = 1.956) between 19 and 25 years old were recruited for the experiment through invitation. The inclusion criterion was that the subjects must be right-handed. The exclusion criteria were color blindness and color weakness. All the subjects had normal or corrected vision. The study was approved by the medical ethics committee of the First People’s Hospital of Xuzhou (Affiliated Hospital of China University of Mining and Technology) (approval document: No. xyy11 [2022] 063), and all the subjects signed an informed consent form. 

### 2.2. Experimental Design

**Figure 1:**
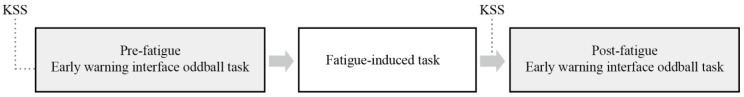
Experimental flow.

Due to the restrictions imposed by the COVID-19 pandemic and other issues, it was not feasible to carry out an actual experiment in a real mine. Therefore, all the experimental tasks in this study were carried out in a lab. The overall experimental process is shown in Figure 1. The experiment includes two early-warning interface oddball tasks and one fatigue-inducing task. The subjects were required to complete the Karolinska sleepiness scale (KSS) before each of the two early-warning interface oddball tasks [35] and remain awake as long as possible before the start of the first early-warning interface oddball task. The experiment was carried out in a quiet environment in the lab. To ensure that the subjects were in a relaxed state, they were reminded to relax, remain in a relatively static position, and blink as few times as possible during the experiment.

#### 2.2.1. Fatigue-Inducing Task

**Figure 2:**
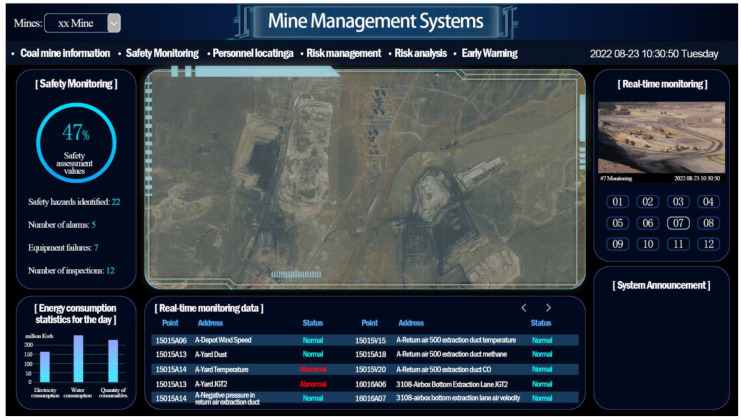
Mine management system’s interface.

The mental fatigue-inducing task was carried out on a mine management system with simulated tasks designed by using E-Prime, a stimulus presentation software. Fatigue was induced by asking subjects to work on the task for 60 min without rest. Figure 2 shows the interface of the mine management system. The mental fatigue-inducing task used a dual-task model. The first task requires the subjects to match which of the four levels of the first page the safety assessment value of the current page belongs to and to respond with the corresponding key. The range of the four levels on the first page was different in each round of the task. The second task was to observe the real-time monitoring data, based on whether the data were abnormal, and perform the corresponding key reaction. Subjects in each round of the experiment had sufficient time memory for the 4 grade ranges, and each round had 20 trials in total. After 60 min, a comparison of the accuracy of the n round task and the 1st/2nd round task was carried out. After the n round task, if KSS score > 6, the subject was considered to have reached the fatigue state; if KSS score ≤ 6 [36], the experiment was continued until the subject was tired. During the experiment, all the judgments of the subjects had to be reported orally to the main subject to prevent the subject from being distracted in the experiment. The EEG and behavior data of the subjects were recorded throughout the fatigue-inducing task.

#### 2.2.2. Oddball Interface Task

The oddball interface task used a 2 × 2 two-factor within-subject design, in which Factor 1 is the mental state of the subjects (alert or fatigued), and Factor 2 is two types of commonly coded visual information on interfaces—color and text.

- Experimental stimulus material

The stimuli used in the experiments are shown in Table 1. The color stimuli use the colors in the five-level warning system in China (see Table 2). According to Chinese standards GB 2893-2008 Safety colors [37], the five colors are: red (255, 0, 0), orange (255, 128, 0), yellow (255, 255, 0), blue (0, 0, 255), and green (0, 209, 12). Aside from color as an important element in interactive interfaces, text is also an important medium for showing information and content. The ways that text is presented, positioned, and rendered affect how meaning is conveyed, readability, legibility, and interaction. Therefore, text was also used as another stimulus. The warning levels were categorized in five levels: I, II, III, IV, and normal. At the same time, cognitive ergonomics research on color and character coding on interfaces indicates that black or blue should be used as the background color for interfaces, and white as the color of interface elements such as text. Therefore, in this study, white text was used on a black background for the interface.

- Experimental process

**Figure 3:**
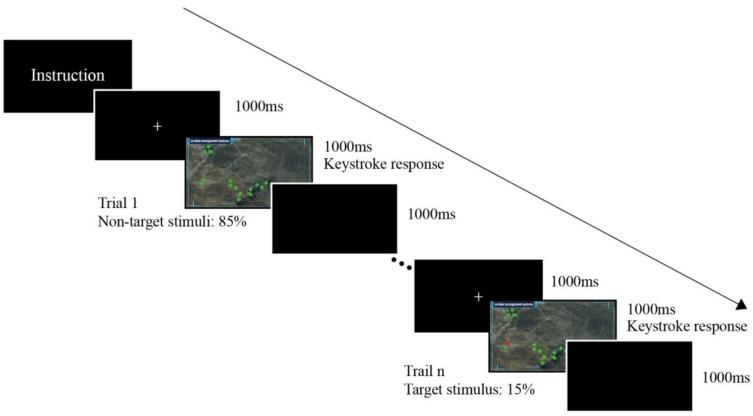
Early-warning interface oddball experiment flow.

**Figure 4:**
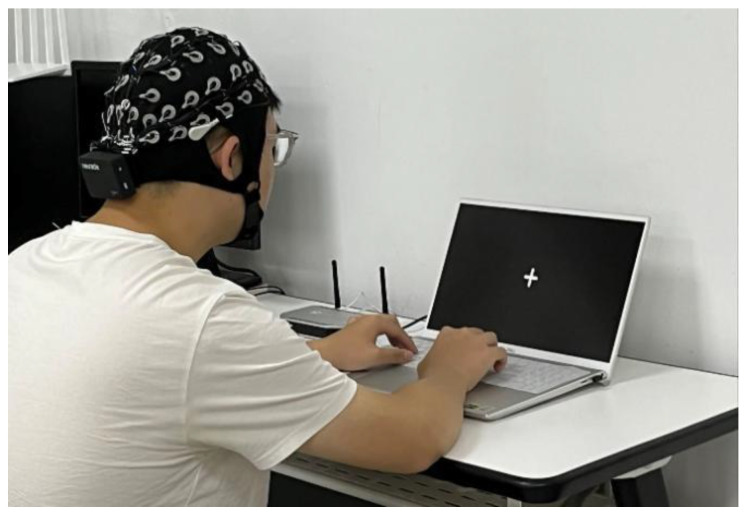
Experimental scenario.

The experiment used the oddball paradigm, in which sequences of stimuli are shown repeatedly until they are interrupted by a deviant stimulus. The deviant stimulus is the early-warning level of I to IV, while the standard stimulus is the normal state on the early-warning interface of the mine management system. The experiment required the subjects to observe images of interface warnings on a screen and choose the response that corresponds to the warning level. The corresponding keys are provided in Table 1. The experimental paradigm was written by using E-Prime, which included two types of conditions (text-coded stimuli × color-coded stimuli). Each type of condition comprised 120 trials, of which each type showed 24 target stimuli, and 96 standard stimuli, with a total of 240 trials (2 × 120 trials). The experimental process is shown in Figure 3. The subjects started the experiment after reading the instructions. Then, they fixated on the “+” sign in the center of the screen as the central fixation point for 1000 ms. Subsequently, a stimulus picture appeared at random for 1000 ms. Afterward, a black screen was presented to give the subject a break for 1000 ms, and then the next trial was presented to the subject. The sequence of stimulus pictures is random, to prevent subjects from forming the habit of prediction. During the experiment, EEG and behavior data (reaction time and accuracy rate) were recorded, respectively. All the stimuli were presented on a 15.6-inch LCD screen with a resolution of 1920 × 1080 pixels and a refresh rate of 60 Hz. The viewing distance between the subjects and the center of the screen was about 550–600 mm. The experimental setup is shown in Figure 4.

### 2.3. EEG Signal Acquisition and Processing

The acquisition of the EEG signals was carried out by using a NeuSen W (Neuracle, Ltd., Changzhou, China) 64-channel EEG acquisition system, and the sampling frequency used was 1000 Hz. The internationally accepted 10–20 system which is used to place the scalp electrodes was used and the scalp resistance was set to be less than 5 kΩ. The raw EEG data were imported into EEGLAB for processing. The main processing steps were: data integration, electrodes’ placement, data filtering, down-sampling, re-referencing, segmentation, baseline correction, EMG removal, eye movement, superimposed averaging, etc., to extract the amplitude and phase of the ERP waveforms.

In this study, the eigenvalues of P200 elicited by the target stimuli were calculated. The marked target stimuli were segmented starting at 200 ms before and ending at 800 ms after the stimulus onset (−200–800 ms). Then, baseline correction was performed on all the segments (200 ms before ending), and the processed EEG data segments were superimposed and averaged. The P200 waveform was obtained, and the ERP waveform was extracted in accordance with the component time-history analysis (150–250 ms). Finally, the eigenvalues of the amplitude and latency of the ERP waveforms were obtained.

### 2.4. Statistical Methods

The user behavior data and EEG eigenvalues were analyzed by using SPSS (IBM Corp., Armonk, NY, USA). Multivariate analysis of variance and repeated measures analysis of variance were used to analyze the behavior data of the subjects in terms of their subjective evaluation of the early-warning interface before and after the fatigue-inducing task and compared to the P200 based on two factors: text vs. color and alert vs. fatigued. Whether the eigenvalues of the amplitude and latency of the ERP waveforms were statistically different was compared, and whether the subjects developed fatigue after the fatigue-inducing task was tested.

## 3. Results

### 3.1. Fatigue Analysis

#### 3.1.1. Subjective Fatigue Analysis

The KSS scale was used to understand the mental fatigue of the subjects. A higher score indicates more subjective fatigue. As shown in Table 3 and according to the statistical results, the KSS scores of the subjects after the fatigue-inducing task were significantly higher than those before the fatigue-inducing task (F = 102.675, *p* = 0 < 0.05). This shows that after the fatigue-inducing task, the subjects were all fatigued. 

#### 3.1.2. Objective Fatigue Analysis

**Figure 5:**
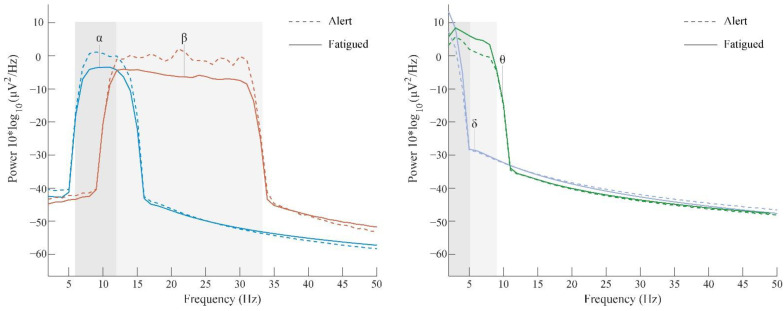
Power spectra of θ, α, δ, and β rhythms before and after fatigue-inducing tasks.

Studies have shown that the EEG signal is decomposed into 4 bands: delta (δ) (0.5–4 Hz), theta (θ) (4–8 Hz), alpha (α) (8–14 Hz), and beta (β) (14–30 Hz) bands, and these can be measured to detect the current mental state [38]. When the subjects were fatigued, the decreased arousal level elevated the delta power [39]. Early stages of fatigue can be indicated by an increase in theta activity [40]. The alpha activity reflects a relaxed wakefulness state and decreases with stimulation or visual fixation [41]. Additionally, beta activity has also been linked to the level of alertness and decreases over a long-term monotonous task [42]. The EEG data of the subjects in the resting state before and after the fatigue-inducing task were obtained, and the theta, alpha, delta, and beta (θ, α, δ, and β rhythms, respectively) frequency bands were extracted from the O1, O2, F3, and F4 channels [43,44]. After preprocessing was complete, the power changes of the θ, α, δ, and β rhythm bands of the subjects before and after the fatigue-inducing task were as shown in Figure 5. It can be observed that the power of the slow frequency (δ and θ) was increased, and the faster frequency was decreased (α and β), thus validating the transition of the subjects from an alert to a fatigued state from a physiological point of view, which is consistent with the results of the subjective fatigue evaluation.

### 3.2. Behavior Data Analysis

**Figure 6:**
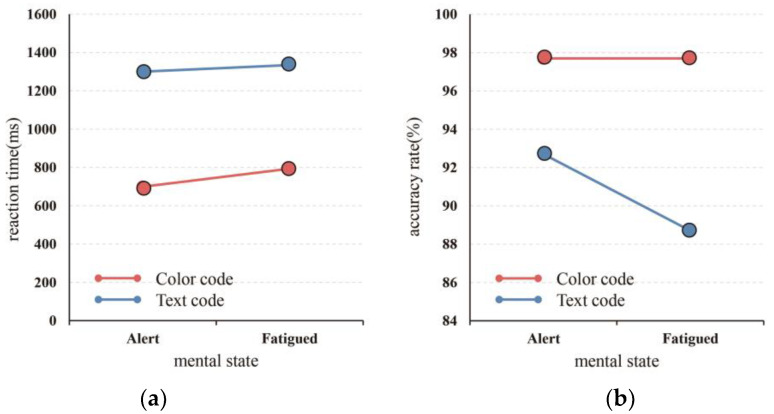
(**a**) Accuracy rate of mental state × visual code. (**b**) Reaction time of mental state × visual code.

A multivariate analysis of variance was performed on the reaction time and accuracy rate of the subjects before and after the fatigue-inducing task, and the results are shown in Table 4 and Table 5 and Figure 6. The results show that the reaction time before and after the fatigue-inducing task was significantly different (F = 5.303, *p* = 0.038 < 0.05). However, the reaction time to the text was significantly higher than to color (F = 5.303, *p* = 0.038 < 0.05). The interaction effect of the mental state and visual code was not significant (*p* > 0.05). These results show that fatigue indeed influenced the reaction time of the subjects, and their reaction time while fatigued was longer. The results also show that the reaction time to color was shorter, that is, the subjects responded more quickly to color as opposed to text. 

As shown in Table 5 and Figure 6b, there was a significant difference in the accuracy rate between the text and color before and after the fatigue-inducing task (F = 26.963, *p* = 0 < 0.05). The accuracy rate of text before and after the fatigue-inducing task was significantly lower than that of color (F = 14.407, *p* = 0.002 < 0.05) and the interaction effect of the mental state and visual code form was significant (*p* = 0 < 0.05). These results indicate that fatigue indeed influenced accuracy, and accuracy while fatigued was reduced. Additionally, the accuracy of color was higher than that of text.

### 3.3. ERP Analysis

**Figure 7:**
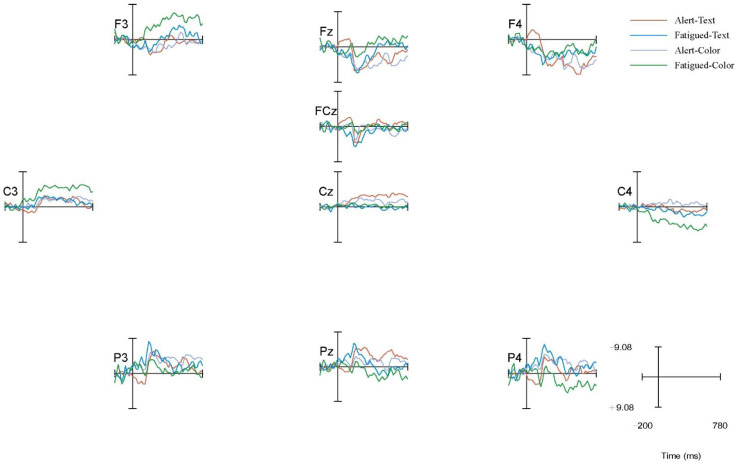
The total average waveform of the oddball paradigm P200.

The EEG data were collected at ten electrode points (F3, Fz, F4, FCz, C3, Cz, C4, P3, Pz, P4) with the use of EEGLAB to understand the cognitive workload at P200. A comparison of the alert vs. fatigued state based on the overall average waveform of P200 in the oddball paradigm is shown in Figure 7.

**Figure 8:**
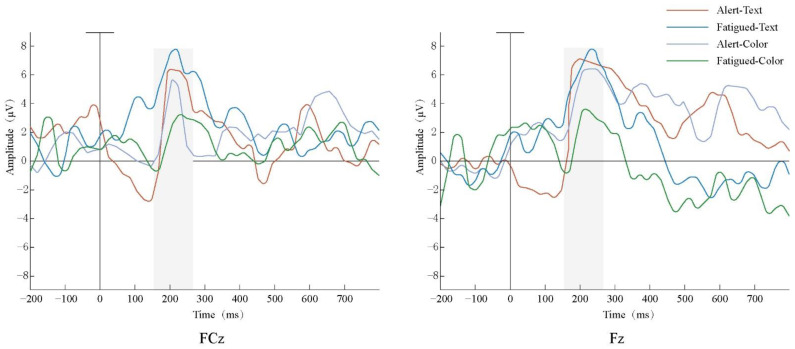
Target stimulation mean ERP in Fz and FCz.

After studying the brain topography along with relevant research theories, it was found that the waveforms of the electrode points in the frontal area (Fz) and frontal central area (Fcz) showed significant changes. Therefore, the two Fz and Fcz electrodes located on the forehead and central frontal area were selected. To examine the characteristics of P200 extracted from each electrode, the superimposed waveform of the total mean EEG induced by the P200 extraction is shown in Figure 8. 

**Figure 9:**
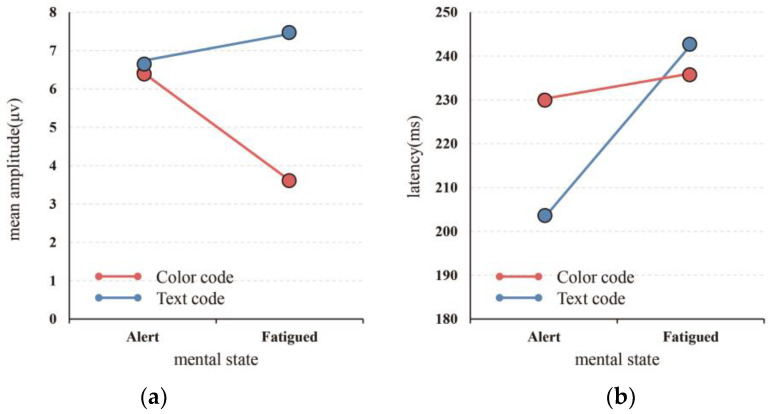
(**a**) Mean amplitude of mental state × visual code. (**b**) Latency of mental state × visual code.

The target stimulus caused a relatively obvious P200 waveform between 150 and 250 ms. Furthermore, the amplitude (μV) and latency (ms) of P200 of the two electrode points at Fz and FCz were measured, and the results are shown in Table 6 and Table 7 and Figure 9. The P200 was intercepted from 150 to 250 ms after the target stimulus was produced, and a repeated ANOVA was performed on the text- or color- and mental state (alert vs. fatigued)-induced P200 amplitude. The results showed that the main effect of visual code (color or text) was significant (F = 24.946, *p* = 0.015 < 0.05), and the main effect of the mental state had no significant difference (*p* = 0.220 > 0.05). The interaction effect of the mental state and visual code was not significant (*p* > 0.05). 

At the same time, a repeated ANOVA was performed on the text- or color- and mental state (alert vs. fatigued)-induced P200 latency. The results showed that the main effect of the mental state was significant (F = 11.820, *p* = 0.041 < 0.05). However, there was no significant difference in the main effect of the visual code (*p* = 0.327 > 0.05). The interaction effect of the mental state and visual code was not significant (*p* > 0.05).

## 4. Discussion

### 4.1. Fatigue Analysis

The analysis of the fatigued state in this study adopted a method that combines both a subjective rating and an objective physiological indicator (EEG). The results showed that the subjects were fatigued after the fatigue-inducing task (KSS score > 6). The physiological results, such as an increase in the power of the δ and θ frequencies and a decrease in the power of the α and β frequencies after the fatigue-inducing task, also demonstrated the shift from an alert state to a fatigued state, which is consistent with the subjectively indicated degree of fatigue. The results are in good agreement, and the robustness of the fatigue-inducing task was verified objectively and quantitatively.

### 4.2. Behavior Data Analysis

The behavioral data showed that the mental state had a significant effect on the reaction time and accuracy rate. The reaction times of the subjects in the fatigued state were longer than those in the alert state, and the accuracy rates in the fatigued state were significantly lower than those in the alert state. Compared with color, the mental state had a greater effect on text, and subjects in the fatigued state had a significantly lower accuracy rate when processing the text-type interface. Visual code had a significant effect on the reaction time and accuracy rate. Probably due to the readability effect of the text and the roman numerals of the abstract increasing the comprehension difficulty of the subjects [45], the reaction time of the text was relatively long, and the accuracy rate was relatively low with respect to color.

### 4.3. ERP Analysis

From the analysis of the ERP characteristics of the oddball paradigm before and after the fatigue-inducing task, the P200 amplitude showed obvious changes, especially in the prefrontal region (FCz and FC), which is consistent with the view that P200 is mainly concentrated in this region [46]. This indicates that in situations where early warnings are given, the prefrontal lobe of the brain will be most alerted by visual stimuli, thereby mobilizing more attention resources, and at the same time, the brain will be in a state of high tension and alertness. 

As can be seen from Figure 8, the amplitude of color when in the alert state was higher than that of the fatigued state. However, for text, the amplitude of the fatigued state was larger than that when the subject was alert. The text stimuli in the fatigued state caused the largest P200 amplitude. It was also found that the amplitude of P200 with text was larger than that with color as the visual code in the different kinds of mental states, which shows that text is more likely to consume attention resources. The text change in the fatigue state was not as intuitive as the color change, and the subjects were relatively distracted, resulting in a higher amplitude of the text. This is because P200 is associated with attention given to target stimuli in the early stages [47], so a larger amplitude denotes more attentional resources consumed. This shows that in designing early-warning interfaces of mine management systems, text consumes more attention resources as opposed to color, the operating load of the subjects is large, and the mental load is also increased. This is consistent with the view that Duchnicky et al. found through eye tracking, that subjects paid almost twice as much attention to the image area as the text area [48], indicating that the non-text interface attracted the attention of the subjects.

From Table 7 and Figure 9, it can be seen that compared to alertness, the latency of text and color was increased in the fatigue state, thus reflecting that the information processing and executive ability to target stimuli were reduced. It can be observed from Figure 9b that when the subjects shifted from an alert to a fatigued state, the increase in the latency of text was greater than that of color, thus indicating that compared with color used as a visual code, information processing and executive ability with text as a visual code were reduced [49]. It may be that compared with text, subjects are more familiar with color, consume fewer cognitive resources, and have a short latency to P200 [17]. This is also validated from the behavioral and physiological perspectives that Christ proposed, that the search performance of color stimuli during search tasks is significantly better than that of text stimuli [50].

The above results all reflect that the mental state has a certain degree of influence on the ability of the subjects to process the early-warning information of a mine management system, and the ability of the subjects to process the early-warning information in the fatigued state was reduced, especially when dealing with text as a visual aid of the early-warning information. The phenomenon is more obvious, which is consistent with the subjective feelings of the subjects, such as difficulties in concentrating and slow reactions in the experiment. 

## 5. Conclusions

The results showed that the target stimulus caused a relatively obvious P200 waveform between 150 and 250 ms, which was mainly found in the prefrontal region. In the fatigued state, the performance of text stimulus was low, the amplitude of P200 was high, the latency was increased, and it incurred the longest response time and the lowest accuracy rate. On the other hand, the color stimulus provided the smallest amplitude and incurred a relatively faster response time, and a higher accuracy rate. In the design of an early-warning interface of mine management systems, text increases the difficulty of the task as opposed to color, so that the operating load of the subjects is larger, and more attention resources are used during the processing of cognitive load. The findings in this study provide the basis for the design of an early-warning interface for mine management systems, to ensure the work efficiency and safety. As with all studies, this paper has its limitations. Only a part of the EEG data of the subjects was examined, and the experimental environment was laboratory-based. In the future, more detailed EEG data should be analyzed, and further research can be performed in actual mines.

## Figures and Tables

**Table 1:** Experimental materials.

Early-Warning Level					
	Normal	Level IV Warning	Level III Warning	Level II Warning	Level I Warning
color code	●	●	●	●	●
text code	Normal	IV	III	II	I
corresponding keys	{SPACE}	l	k	s	a

**Table 2:** Five-level warning system.

Level	Color	Definition
I	Red	Alarm
II	Orange	Vigilance
III	Yellow	Caution
IV	Blue	Attention
Normal	Green	Secure

**Table 3:** Correctness and reaction time before and after fatigue-inducing tasks.

	Before Fatigue-Inducing Task	After Fatigue-Inducing Task
KSS Score	2.5714 ± 0.787	7.857 ± 11.107

**Table 4:** Multivariate ANOVA results for reaction times of mental state and visual code.

	Value	F	df	*p*
mental state	0.710	5.303	13	0.038
visual code	0.014	890.809	13	0.000
mental state × visual code	0.809	3.061	13	0.104

**Table 5:** Multivariate ANOVA results for accuracy rates of mental state and visual code.

	Value	F	df	*p*
mental state	0.325	26.963	13	0.000
visual code	0.474	14.407	13	0.002
mental state × visual code	0.325	26.963	13	0.000

**Table 6:** Multivariate ANOVA results for P200 mean amplitude of mental state and visual code.

	Value	F	df	*p*
mental state	0.557	2.387	13	0.220
visual code	0.107	24.946	13	0.015
mental state × visual code	0.263	8.386	13	0.063

**Table 7:** Multivariate ANOVA results for P200 latency of mental state and visual code.

	Value	F	df	*p*
mental state	0.202	11.820	13	0.041
visual code	0.688	1.363	13	0.327
mental state × visual code	0.305	6.832	13	0.079

## Data Availability

The data presented in this study are available upon request from the corresponding author.

## References

[1] Deng M., Chan A.H.S., Wu F., Sun L. (2018). Depth perception, dark adaptation, vigilance and accident proneness of Chinese coal mine workers. Int. J. Occup. Saf. Ergon..

[2] Li X., Cao Z., Xu Y. (2021). Characteristics and trends of coal mine safety development. Energy Sources Part A Recovery Util. Environ. Eff..

[3] Wang S., Li S., Yang K., Feng Y., Liu S., Zhang J., Cao Y., Bai Z. (2022). Research on Adaptive Management of the Social–Ecological System of a Typical Mine–Agriculture–Urban Compound Area in North Shanxi, China. Int. J. Environ. Res. Public Health.

[4] Singo J., Isunju J.B., Moyo D., Bose-O’Reilly S., Steckling-Muschack N., Mamuse A. (2022). Accidents, Injuries, and Safety among Artisanal and Small-Scale Gold Miners in Zimbabwe. Int. J. Environ. Res. Public Health.

[5] Xu R., Luo F. (2021). Risk prediction and early warning for air traffic controllers’ unsafe acts using association rule mining and random forest. Saf. Sci..

[6] Yan K., Shao J., Zhu Z., Zhang K., Yao J., Tian F. (2021). Display interface design for rollers based on cognitive load of operator. J. Soc. Inf. Disp..

[7] Ya-Feng N., Jin L., Jia-Qi C., Wen-Jun Y., Hong-Rui Z., Jia-Xin H., Lang X., Jia-Hao W., Guo-Rui M., Zi-Jian H. (2022). Research on visual representation of icon colour in eye-controlled systems. Adv. Eng. Inform..

[8] Mallick Z., Badruddin I.A. (2007). Optimization of operating parameters of video display units in text reading task: Luminance contrast, viewing distance, and character size. J. Soc. Inf. Disp..

[9] Faiola T., DeBloois M.L. (1988). Designing a visual factors-based screen display interface: The new role of the graphic technologist. Educ. Technol..

[10] Rolke B., Festl F., Seibold V.C. (2016). Toward the influence of temporal attention on the selection of targets in a visual search task: An ERP study. Psychophysiology.

[11] Yeh Y.-Y., Lee D.-S., Ko Y.-H. (2013). Color combination and exposure time on legibility and EEG response of icon presented on visual display terminal. Displays.

[12] Bayliss J., Ballard D. (1998). Single Trial P300 Recognition in a Virtual Environment.

[13] Chen M., Xue C., Wang H., Chen Y., Li J. (2014). Study of the product color’s image based on the event-related potentials. Proceedings of the 2014 IEEE International Conference on Systems, Man, and Cybernetics (SMC).

[14] Xue C., Wu X., Niu Y., Zhou L., Shao J., Shen Z., Stephanidis C. (2015). Brain Mechanism Research on Visual Information Cognition of Digital Human Computer Interface. HCI International 2015—Posters’ Extended Abstracts. HCI 2015.

[15] Liu B., Meng X., Wu G., Huang Y. (2012). Feature precedence in processing multifeature visual information in the human brain: An event-related potential study. Neuroscience.

[16] Misra M., Holcomb P.J. (2003). Event–related potential indices of masked repetition priming. Psychophysiology.

[17] Pernet C., Basan S., Doyon B., Cardebat D., Demonet J.-F., Celsis P. (2003). Neural timing of visual implicit categorization. Cogn. Brain Res..

[18] Barber H., Vergara M., Carreiras M. (2004). Syllable-frequency effects in visual word recognition: Evidence from ERPs. NeuroReport.

[19] Huang Y.-X., Luo Y.-J. (2006). Temporal course of emotional negativity bias: An ERP study. Neurosci. Lett..

[20] Polezzi D., Lotto L., Daum I., Sartori G., Rumiati R. (2008). Predicting outcomes of decisions in the brain. Behav. Brain Res..

[21] Gajewski P.D., Ferdinand N.K., Kray J., Falkenstein M. (2018). Understanding sources of adult age differences in task switching: Evidence from behavioral and ERP studies. Neurosci. Biobehav. Rev..

[22] Sur S., Sinha V.K. (2009). Event-related potential: An overview. Ind. Psychiatry J..

[23] Luck S.J., Hillyard S.A. (1994). Electrophysiological correlates of feature analysis during visual search. Psychophysiology.

[24] Schindler S., Kissler J. (2016). Selective visual attention to emotional words: Early parallel frontal and visual activations followed by interactive effects in visual cortex. Hum. Brain Mapp..

[25] Jin J., Zhang W., Chen M. (2017). How consumers are affected by product descriptions in online shopping: Event-related potentials evidence of the attribute framing effect. Neurosci. Res..

[26] Mun S., Kim E.-S., Park M.-C. (2014). Effect of mental fatigue caused by mobile 3D viewing on selective attention: An ERP study. Int. J. Psychophysiol..

[27] Lee H.-J., Kim L., Kim Y.-K., Suh K.-Y., Han J., Park M.-K., Park K.-W., Lee D.-H. (2004). Auditory Event-Related Potentials and Psychological Changes during Sleep Deprivation. Neuropsychobiology.

[28] Sun L., Guo Z., Yuan X., Wang X., Su C., Jiang J., Li X. (2022). An Investigation of the Effects of Brain Fatigue on the Sustained Attention of Intelligent Coal Mine VDT Operators. Int. J. Environ. Res. Public Health.

[29] Conley E.M., Michalewski H.J., Starr A. (1999). The N100 auditory cortical evoked potential indexes scanning of auditory short-term memory. Clin. Neurophysiol..

[30] Miller M.W., Rietschel J.C., McDonald C.G., Hatfield B.D. (2011). A novel approach to the physiological measurement of mental workload. Int. J. Psychophysiol..

[31] Moore T.M., Key A.P., Thelen A., Hornsby B. (2017). Neural mechanisms of mental fatigue elicited by sustained auditory processing. Neuropsychologia.

[32] Dimitrakopoulos G.N., Kakkos I., Dai Z., Wang H., Sgarbas K., Thakor N., Bezerianos A., Sun Y. (2018). Functional Connectivity Analysis of Mental Fatigue Reveals Different Network Topological Alterations Between Driving and Vigilance Tasks. IEEE Trans. Neural Syst. Rehabil. Eng..

[33] Zhang H., Lu W., Tian F., Dang Y., Tian S., Xu J., Yang P., Chen L. (2021). The ERP characteristics of miners fatigue. J. Xi’an Univ. Sci. Technol..

[34] Huang Y., Li H., Yang Y., Wang J., Jansz J. (2021). Event-Related Potential Sensing Analysis on the Risk Perception and Decision-Making by Grassroots Managers in Different Fatigue States. J. Healthc. Eng..

[35] Åkerstedt T., Gillberg M. (1990). Subjective and Objective Sleepiness in the Active Individual. Int. J. Neurosci..

[36] Martensson H., Keelan O., Ahlstrom C. (2019). Driver Sleepiness Classification Based on Physiological Data and Driving Performance from Real Road Driving. IEEE Trans. Intell. Transp. Syst..

[37] (2008). Safety Color.

[38] Sengupta A., Tiwari A., Routray A. (2017). Analysis of cognitive fatigue using EEG parameters. Proceedings of the 2017 39th Annual International Conference of the IEEE Engineering in Medicine and Biology Society (EMBC).

[39] Guo W., Ren J., Wang B., Zhu Q. (2015). Effects of Relaxing Music on Mental Fatigue Induced by a Continuous Performance Task: Behavioral and ERPs Evidence. PLoS ONE.

[40] Fan X., Zhou Q., Liu Z., Xie F. (2015). Electroencephalogram assessment of mental fatigue in visual search. Bio-Med. Mater. Eng..

[41] Li J., Li H., Wang H., Umer W., Fu H., Xing X. (2019). Evaluating the impact of mental fatigue on construction equipment operators’ ability to detect hazards using wearable eye-tracking technology. Autom. Constr..

[42] Jap B.T., Lal S., Fischer P., Bekiaris E. (2009). Using EEG spectral components to assess algorithms for detecting fatigue. Expert Syst. Appl..

[43] Lee Y.-J., Kim H.-G., Cheon E.-J., Kim K., Choi J.-H., Kim J.-Y., Kim J.-M., Koo B.-H. (2019). The Analysis of Electroencephalography Changes Before and After a Single Neurofeedback Alpha/Theta Training Session in University Students. Appl. Psychophysiol. Biofeedback.

[44] Potts G.F., Patel S.H., Azzam P.N. (2004). Impact of instructed relevance on the visual ERP. Int. J. Psychophysiol..

[45] Ali A.Z.M., Wahid R., Samsudin K., Idris M.Z. (2013). Reading on the Computer Screen: Does Font Type have Effects on Web Text Readability?. Int. Educ. Stud..

[46] Yue K., Wang D., Hu H., Fang S. (2018). The correlation between visual fatigue and duration of viewing as assessed by brain monitoring. J. Soc. Inf. Disp..

[47] Lijffijt M., Lane S.D., Meier S.L., Boutros N.N., Burroughs S., Steinberg J.L., Moeller F.G., Swann A.C. (2009). P50, N100, and P200 sensory gating: Relationships with behavioral inhibition, attention, and working memory. Psychophysiology.

[48] Duchnicky R.L., Kolers P.A. (1983). Readability of Text Scrolled on Visual Display Terminals as a Function of Window Size. Hum. Factors..

[49] Tsang S.N.H., Chan A.H.S., Pan X., Man S.S. (2021). Auditory versus visual spatial stimulus-response mappings in tracking and discrete dual task performance: Implications for human-machine interface design. Ergonomics.

[50] Christ R.E. (1975). Review and analysis of color coding research for visual displays. Hum. Factors.

